# An Effective Cathartic

**Published:** 1879-09

**Authors:** 


An Effective Cathartic.—The hearty welcome which is extended by all truly scientific medical men to important advances in therapeutics is our excuse for relating the following:

The town of B———, in this State, has two medical men; one a regular graduate, the other a modest "vet" whose knowledge of cow and horse disease is briefly summed up in the portentous words "great experience." Mr. G—— was taken suddenly ill. The regular physician was out of town and not expected to return until the next day. After much solicitation at the hands of the sick man's friends, the "vet" consented to see Mr. G., and prescribe for him. After looking him over carefully he delivered himself as follows:

"Wall if he was a cow, I should reckon he had an allfired attack of billious collic, and I sort a guess I'd give that cow a pint of castor ile and a pound roshelle salts. Seein, as how Mr. G. ain't a cow, I'd sort a reduce the dose, giving him half a pint a ile and quarter a pound of salts. It's the only thing that'll fetch him."

The friends accordingly forced as much of this really valuable prescription into the sufferer as was possible, he being only semi-conscious.

The following morning the "vet" called to see his patient, and asked—"Well! did the medicine have the desired result—did it move him?"

"It most certainly did," said a red-eyed friend.

"How often?" asked the "vet"!

"Four times—*once before death and three times afterwards!*"—*Hosp. Gazette.*

